# The Therapeutic Potential of Adipose Tissue-Derived Mesenchymal Stem Cells to Enhance Radiotherapy Effects on Hepatocellular Carcinoma

**DOI:** 10.3389/fcell.2019.00267

**Published:** 2019-11-12

**Authors:** Lingyun Wu, Qiuying Tang, Xin Yin, DanFang Yan, Mengmeng Tang, Jiaojiao Xin, Qiaoling Pan, Chiyuan Ma, Senxiang Yan

**Affiliations:** ^1^Department of Radiation Oncology, The First Affiliated Hospital, Zhejiang University School of Medicine, Hangzhou, China; ^2^Department of Pathology, The First Affiliated Hospital, Zhejiang University School of Medicine, Hangzhou, China; ^3^State Key Laboratory for Diagnosis and Treatment of Infectious Diseases, Collaborative Innovation Center for Diagnosis and Treatment of Infectious Diseases, The First Affiliated Hospital, Zhejiang University School of Medicine, Hangzhou, China; ^4^Department of Orthopaedic Surgery, The Second Affiliated Hospital, Zhejiang University School of Medicine, Hangzhou, China

**Keywords:** adipose tissue-derived mesenchymal stem cells, radiotherapy, hepatocellular carcinoma, interferon-induced transmembrane 1, combination therapy

## Abstract

Several studies have investigated strategies to improve the clinical efficacy of radiotherapy (RT) against hepatocellular carcinoma (HCC), yet the prognosis remains poor. Human adipose tissue-derived mesenchymal stem cells (AT-MSCs), easily accessible and abundant in quantity, have represented as an attractive therapeutic tool for the stem cell-based treatment for cancer diseases. Through direct co-culture and indirect separate culture experiments, we showed that AT-MSCs could enhance inhibitory effect of RT on reducing HCC cell growth, migration and invasion in both *in vitro* and *in vivo* experiments. RNA-sequencing analysis revealed a noticeable interferon-induced transmembrane 1 (IFITM1)-induced tumor gene signature. Gain and loss of mechanistic studies indicated that mechanism was attributed to downregulated expression of signal transducer and activator of transcription 3 (STAT3) and matrix metallopeptidases (MMPs) and upregulated expression of P53 and caspases. Collectively, our findings suggest that AT-MSCs might enhance the therapeutic effects of RT on HCC, providing a rationale for AT-MSCs and RT combination therapy as a new remedy for HCC.

## Introduction

Liver cancer is the fourth leading cause of cancer mortality worldwide, with 782,000 deaths annually, estimated through the GLOBOCAN database 2018 (Bray et al., 2018). Primary liver cancer consists of hepatocellular carcinoma (HCC) (approximately 90% of all cases) and intrahepatic cholangiocarcinoma as well as other rare types. Because of the poor treatment efficacy, early metastasis and strong invasive activity, the overall survival and 5-year disease-free survival of HCC patients remain low. For patients with advanced HCC who are not candidates for surgical resection or transplantation, radiotherapy (RT) is commonly used as a potential treatment option. Liver-direct radiation therapy include external beam radiation therapy (EBRT) and stereotactic body radiation therapy (SBRT) (Benson et al., 2017). These two protocols consist of high-dose radiation (HDR), with the former ranging from 1.8 to 2.0 Gy per fraction and the later 6–15 Gy. However, the rates of local recurrence and distant metastasis remain unsatisfactory despite precise high-dose irradiation. Cell survival assays, largely used in radiobiological studies, have provided evidence that cells be induced to radiation exposure do not die immediately (Chu et al., 2002). Contact of surviving tumor and non-tumor cells with the internal and external environments via diverse signaling pathways and/or gene expression pathways leads to production of various chemokines, cytokines, growth factors, and protein hormones. The mechanism of how these close-knit cellular interactions affect the tumor response to radiation is currently an important area of study. In non-tumor cells, mesenchymal stem cells (MSCs) take an essential part. Recent studies have suggested MSCs have an inhibitory effect on HCC, suggesting that MSCs have potential as a novel therapeutic agent (Qiao et al., 2008b; Li et al., 2013).

Using experimental models of Lewis lung carcinoma and melanoma cell lines, Maestroni et al. (1999) first reported the ability of MSCs to inhibit primary tumor growth (Maestroni et al., 1999). Since then, MSCs have been shown to suppress a variety of tumor types via regulation of cellular signaling, cell cycle progression, apoptosis induction, and immune cell engraftment (Qiao et al., 2008a; Klopp et al., 2011). Adipose tissue-derived mesenchymal stem cells (AT-MSCs) are one of the most promising types of MSCs, which can be easily obtained by minimally invasive procedures and can differentiate into numerous cell lineages (Wu et al., 2018). Due to recent advancements in anticancer treatments, interests in the role of AT-MSCs in combating malignant diseases has grown (Lu et al., 2018; Jung et al., 2019). We are interested to determine whether AT-MSCs can be used as a synergistic treatment with RT on HCC cells, along with the underlying molecular mechanism.

In the present study, we have performed multiple experiments to evaluate the anti-cancer potential of AT-MSCs combined with RT on HCC cell lines. To the best of our knowledge, this is the first study to evaluate the combination effectiveness between these two treatments. After verifying our RNA sequencing results, we determined that AT-MSCs enhanced the efficacy of RT on tumor regression via inhibition of interferon-induced transmembrane 1 (IFITM1) gene expression. Our data illustrate the therapeutic potential of human AT-MSCs on enhancing the treatment effects of RT on HCC.

## Materials and Methods

### Mesenchymal Stem Cell Isolation, Culture, and Characterization

Human AT-MSCs were isolated from the lipoaspirates of healthy donors undergoing elective liposuction. All donors were 25–50 years of age and provided informed consent for the use of their adipose tissue. Adipose tissue was harvested according to a protocol approved by the Clinical Research Ethics Committee of the First Affiliated Hospital, Zhejiang University School of Medicine (Pittenger et al., 1999). Donors with malignancies or systemic or infectious diseases were excluded. AT-MSCs were isolated and cultured as in previously described protocols (Lee et al., 2015). Once the AT-MSCs reached 70–80% confluence, the adherent cells were detached using trypsin/EDTA and passaged at a 1:3 dilution. For our experiment, we used cells from passages 3–6. For further investigation, the medium was refreshed once the cells had entered the logarithmic growth phase. Detailed materials and reagents are available in Supplementary Tables S1, S2.

**FIGURE 1:**
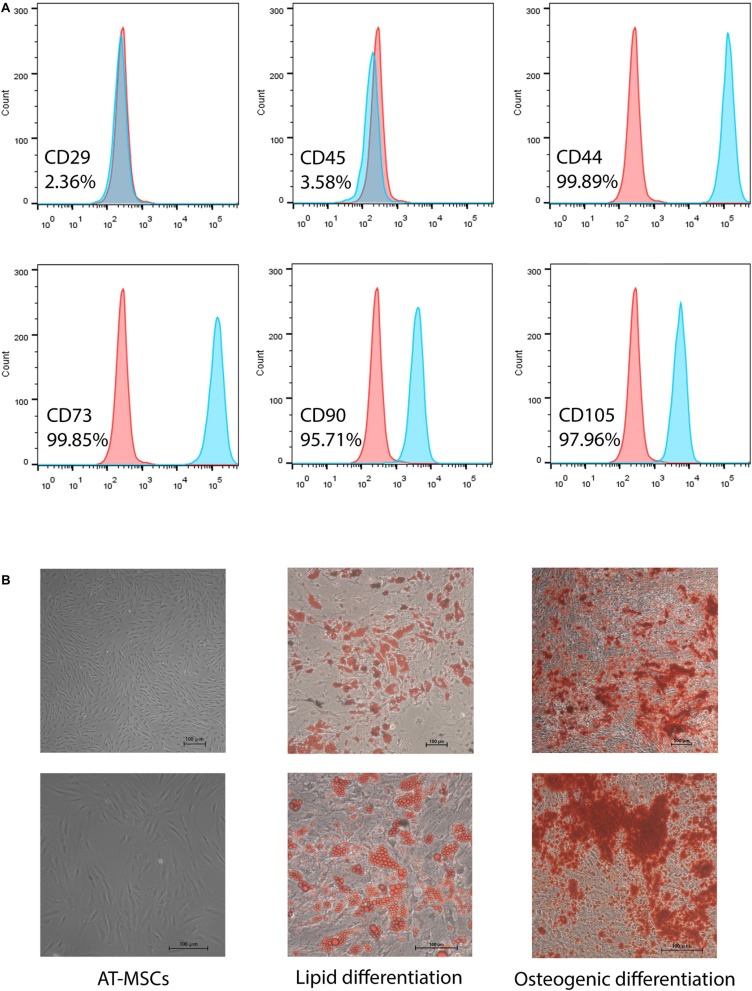
Characterization of human adipose-tissue derived mesenchymal stem cells (AT-MSCs). **(A)** Representative of flow cytometry results of MSC markers: CD44, CD45, CD73, CD90, and CD105. **(B)** Morphology of AT-MSCs (left). Representative images of AT-MSCs differentiated into adipocytes and osteocytes (middle and right). Adipocytes and osteocytes were stained by Oil Red O and Alizarin red, respectively. Scale bar 100 μm.

Adipose tissue-derived mesenchymal stem cells were identified as being MSCs by morphology, phenotypic identification and the ability of inducing into osteogenic and adipogenic differentiation (Figure 1).

### Tumor Cell Lines Culture

Two human HCC lines HepG2 were obtained from the American Type Culture Collection (ATCC, Manassas, VA, United States). HCC cell line HuH7 and one normal liver cell line (LO2) used in this study were generous gifts from Dr. Wang Weilin (Zhejiang University, China). All of the cells were maintained in a humid atmosphere containing 5% CO_2_ at 37°C and were passaged using standard cell culture techniques. STR profiles were used to ensure the quality and integrity of the cell lines (Supplementary Data Sheets S1, S2).

### Animal Models

Male Balb/c nude mice (5- to 6-week-old) were purchased from the Shanghai Experimental Animal Center, Chinese Academy of Sciences, for the establishment of the mouse model bearing human HCC Huh7 xenografts. According to data from ATCC, this cell lines is tumorigenic in nude mice. The mice were housed under aseptic conditions and were provided autoclaved rodent diet and sterile water. All of the mouse studies were conducted in accordance with the National Institute Guide for the Care and Use of Laboratory Animals. The experiments were conducted as per protocol approved by the local hospital ethics committee.

Human HCC Huh7 cells were grown to 80% confluence in 90 mm tissue culture dishes. After harvesting, the cells were resuspended in saline at 4°C to a final concentration of 1 × 10^7^ cells/mL. The right flank of 5-week-old Balb/c nude mice was injected subcutaneously with 100 μL cell suspension (1 × 10^6^ cells) via a disposable syringe on day-14. After the tumor volume reached ∼100 mm^3^ in volume at day 0 after implantation, the animals were randomized into four groups. (1) In the control group (CTRL), tumors on the right flank were injected intravenously injected with saline, handled in exactly the same manner as the mice irradiated or injected with AT-MSCs, although this group did not receive either radiation or AT-MSCs therapy. (2) In the radiotherapy group (RT), the tumor on the right flank was treated with irradiation on day 0. Radiation was delivered by a linear accelerator (Varian Trilogy, Palo Alto, CA, United States) at a dose energy of six MV X-ray and dose rate of 500 MU/min. The mouse tumors were placed directly under an 8 mm thick steel sheet with a 10 mm diameter hole; the rest of the mouse body of each mouse was protected by the steel sheet. (3) In the AT-MSCs group (MSC), tumors were administrated 1 × 10^6^ AT-MSCs suspended in 100 μL saline via the tail vein on day 0. (4) In the Radiotherapy combined AT-MSCs group (RTM), tumors were treated with 1 × 10^6^ AT-MSCs suspended in 100 μL saline injected via the tail vein on day 0 immediately after RT treatment.

Tumor growth and body weight were monitored and recorded every 3 days. Two perpendicular axes (major axis = *a*, minor axis = *b*) of the tumors were measured using calipers, and the tumor volume was calculated using the following formula: *V* = π × *a* × *b*/6. The animals were allowed to rest for 3–4 days before the experiments were finalized. The mice were sacrificed by CO_2_ inhalation once their tumors reached the 1500 mm^3^.

### Histological Evaluation

For histopathologic and immunohistochemical (IHC) analyses, the tumor samples were fixed in 10% neutral buffered formalin, embedded in paraffin blocks and sectioned (5 μm), and stained with hematoxylin and eosin. The sections were randomly numbered prior to observation by an experienced pathologist. Images were acquired using the NanoZoomer 2.0-RS scanner (Hamamatsu Photonics KK, Hamamatsu City, Japan) equipped with scanner software.

The expression of Ki-67 and IFITM1 were evaluated after IHC staining using specific antibodies. Apoptotic cells were detected by TUNEL staining and a DAB Substrate Kit according to the manufacturer’s instructions. The number of positive cells was counted and recorded in four randomly selected high−power fields (magnification, 100×) per section.

### RNA Sequencing

Freshly isolated tumor samples were suspended in Trizol reagent, and the total RNA was immediately extracted according to the manufacturer’s instructions. Total RNA was stored at −80°C for subsequent testing. RNA degradation and contamination were monitored by agarose gels and purity was assessed using the NanoPhotometer^®^ spectrophotometer (Implen, Westlake Village, CA, United States). A sequencing library was prepared using the NEBNext^®^ Ultra^TM^ RNA Library Prep Kit for Illumina^®^ (NEB, Ipswich, MA, United States) following the manufacturer’s recommendations. The library was sequenced using the Illumina HiSeq 2000 and the quality was assessed by the Agilent Bioanalyzer 2100 system (Agilent Tech. Inc., Santa Clara, CA, United States).

### Quantitative Real-Time Polymerase Chain Reaction Analysis

Total RNA was extracted from fresh tumor tissues and cultured cells using Trizol reagent according to the manufacturer’s instructions. Reverse transcription into complementary DNA (cDNA) was performed using the QuantiTect Reverse Transcription kit. Platinum SYBR Green qPCR SuperMix UDG was used for qRT-PCR reactions. The sequences of the specific gene primers (Sangon, Shanghai, China) used for the experiments were as follows: IFITM1 forward, 5′-AGCCAGAAGATGCAC AAGGA-3′ and reverse, 5′-GATCACGGTGGACCTTG GAA-3′; GAPDH forward, 5′-GAAGGTCGGTGTGAACGGA TTTG-3′ and reverse, 5′-CATGTAGACCATGTAGTTG AGGTCA-3′. The median cycle threshold and the fold change in expression at each time point post-treatment relative to the CTRL group was calculated for each gene.

### Western Blot Analysis

Protein samples from tumor cells in the four treatments groups were separated by 12% SDS-PAGE and transferred to nitrocellulose membranes (GE Healthcare, Little Chalfont, United Kingdom). Membranes were blocked and then incubated with the appropriate primary antibodies against the following proteins: IFITM1, STAT3, cleaved-Caspase3, Caspase3, Caspase7, mmp2, mmp9, p53, P21, β-actin. Subsequently, the membrane was incubated with a horseradish peroxidase-conjugated secondary antibody for 1 h at room temperature. The probed proteins were detected using SuperSignal West Femto Maximum Sensitivity Substrate.

### Cell Viability Assay

Harvest of conditioned medium (CM) from AT-MSCs: AT-MSCs were cultured in T175 flasks after reaching approximately 70–80% confluency, followed by removal of 10% FBS. After washing with PBS, fresh serum-free RPMI containing 1% PS was added to the cells for conditioning. CM was harvested at various time points to screen for its effect on tumor cell viability. The CM obtained at 48 h showed the maximum reduction in tumor cell viability, which hereafter referred to as AT-CM, and was utilized for subsequent studies. AT-CM was centrifugated at 2000 × *g* for 15 min, followed by filtration through a 0.22 μm membrane to remove any cell debris, and used undiluted in further experiments.

Hepatocellular carcinoma cells were seeded into 96-well plastic Falcon Petri dishes at a plating density of 3 × 10^3^ cells/well. After 24 h of incubation, the growth medium was removed and replaced with non-conditioned control medium in the CTRL group, non-conditioned control medium followed by treatment with different doses of radiation (5, 10, 15, and 20 Gy) in the RT group, AT-CM in the MSC group, or treated with different doses of radiation (5, 10, 15, and 20 Gy) followed by replacement with AT-CM in the RTM group. After incubation for 12, 24, 48 or 60 h, cell proliferation was analyzed by using CCK8 quantitative colorimetric assay according to the manufacturer’s instructions. The absorbance was measured at 450 nm using a microplate reader (Spectra MAX M3; Molecular Devices, Sunnyvale, CA, United States). AT-CM was aspirated and added with 100 μl of the detergent reagent. A microplate ELISA reader (Biocompare, South San Francisco, CA, United States) was used to measure absorbance at 540 nm, following the manufacturer’s instructions.

### Colony Formation Assay

Hepatocellular carcinoma cells (500/well) were seeded into six-well dishes and treated with different doses of radiation (5, 10, 15, and 20 Gy) following with treatment with AT-CM or non-conditioned control medium and incubated for 7–14 days. Cell colonies were fixed with 70% ethanol, stained with crystal violet (0.5% w/v), and counted. The colonies consisted of at least 50 cells and were visible to the naked eyes. Results are presented as means ± standard deviation (SD) of three independent experiments, with duplicate samples assessed for each treatment condition.

### Co-cultures of AT-MSCs and HCC Cell Colonies

Huh7 cells were seeded as before. HCC cell-formed colonies were treated with irradiation, non-irradiated AT-MSC, co-cultured with AT-MSC after irradiation or left untreated for 7–14 days. Cell colonies were washed, fixed with 70% ethanol and stained with crystal violet. Results are presented as means ± SD of three independent experiments, with duplicate samples assessed for each treatment condition.

### Sphere Formation Assay

Hepatocellular carcinoma cells were seeded into six-well plastic Falcon Petri dishes. After 24 h of incubation, the growth medium was removed and replaced with non-conditioned control medium in the CTRL group, non-conditioned control medium followed by treatment with irradiation in the RT group, AT-CM in the MSC group, or treated with irradiation followed by replacement with AT-CM in the RTM group. After incubation for 48 h, HCC cell lines were cultured and serially plated on an ultra-low attachment six-well plate at 500 cells/well in serum-free DMEM/F-12 supplemented with 20 ng/mL of EGF, 10 ng/mL of bFGF, and B27 supplement for 14 days according to published protocols (Leung et al., 2010). The experiment was conducted as three independent replicates.

### Migration and Invasion Assay

Cell migration and invasion were analyzed *in vitro* using the Transwell insert system (Corning, United States) with or without Matrigel coating (BD, United States), respectively. Medium (600 μL) containing 10% FBS was added outside of the Transwell culture insert. For CTRL group, 100 μL of serum-free medium containing 2 × 10^4^ cells was added to each well of the insert. For RT group, cells were treated with irradiation followed by serum-free medium, then added to the insert. For MSC group, cells treated with serum-free AT-CM were added. For RTM group, cells were treated with irradiation followed by serum-free AT-CM. After incubation for 24 h, the Transwells were washed and cleaned using cotton swabs and then fixed with 100% methanol for 15 min, washed twice with PBS, stained with 0.1% of crystal violet for 10 min, and observed under a microscope (Leica, Germany). The experiment was performed in triplicate.

### Wound Healing Assay

Equal numbers of Huh7 or HepG2 cells were seeded in a 48-well plate. Once the cells reached 90% confluence, a single wound was made by gently scratching the attached cells using a 200 μL sterile plastic pipette tip. Debris was removed by washing the cells with serum-free medium. Immediately or 24 h after incubation at 37°C, phase-contrast images of the cells were obtained digitally. The cells that migrated or extended protrusion from the borders of the wound were quantified in three randomly selected areas per well. The experiment was performed in triplicates.

### Immunofluorescence Analyses

Cells were cultured on six-well Merck Millicell EZ slides (Merck Millipore, Darmstadt, Germany) and allowed to attach overnight. After treatment, the slides were washed, fixed in 4% formaldehyde and permeabilized with 0.5% Triton X-100 in PBS, and stained with primary antibodies against IFITM1, STAT3 for 1 h. After washing three times, cells were stained with secondary antibody-conjugated fluorochromes for 1 h, and the nuclei were stained with 4,6-diamidino-2-phenylindole (DAPI). Images were captured using the Leica SP5 II confocal laser-scanning microscope with Plan-Apochromat 63×/1.4 oil objective and LAS/AF Lite software for analysis.

### Short Hairpin RNA (shRNA) Lentiviral Transduction

Complementary DNA of the full-length human IFITM1 gene was cloned into an expression plasmid pCMV3-FZD8-Flag (Biolink Biological Inc.). Two lentivirus-based shRNAs against IFITM1 gene was designed by Biolink Biotechnology Co., Ltd. (Shanghai, China) to silence IFITM1 mRNA expression (the targeted human IFITM1 sequences are: (IFITM1 shRNA-1) 5′-CCTCATGACCATTGGATTCAT-3′ and (IFITM1 shRNA-2) 5′-CCTGTTCAACACCCTCTTCTT-3′, respectively), and control (non-silencing) shRNA (in Puromycin-C-RS vector). Huh7 cells were plated in six-well plates with fresh medium without antibiotics. When the cells reached 30–50% confluence, they were seeded in serum-free medium and lentivirus particles were added to the culture medium at a multiplicity of infection of 30. Cells were grown at 37°C for 24 h following transfection and then placed in fresh medium. Transfected cells were re-plated in 6 cm dishes and selected for using puromycin (10 μg/mL).

### Statistical Analysis

All experiments were confirmed in at least three independent experiments. Statistical analyses were performed using SPSS 24.0 (SPSS, Chicago, IL, United States) and GraphPad (Prism 9.0, GraphPad Software, San Diego, CA, United States). Quantitative data were expressed as means ± SD otherwise denoted. A one-way analysis of variance (ANOVA) and Student’s *t*-tests were used to determine the statistical significance of the data obtained and to compare the means between groups. *p* < 0.05 was considered statistically significant and all tests were two-sided. In figures, ^∗^*P* < 0.05, ^∗∗^*P* < 0.01, and ^∗∗∗^*P* ≤ 0.001.

## Results

### RT Combined AT-MSCs Treatment Suppresses Tumor Cell Proliferation, Migration, and Invasion

Human adipose-derived MSCs were obtained from lipoaspirates from several healthy women undergoing selective suction-assisted lipectomy and were pooled for all experiments in this research. Immunophenotyped of AT-MSCs after 2–3 passages in culture was following: positive for CD44 + (99.89%), CD73 + (99.85%), CD90 + (95.71%), CD105 + (97.96%), whereas negative for CD29-(2.36%) and CD45-(3.58%) (Figure 1A). These features are consistent with previous findings (Cao et al., 2005). Moreover, multipotential differentiation results showed that adipocytes differentiated from AT-MSCs were positive for oil red O staining with visible lipid droplets, and AT-MSC-differentiated osteocytes were positive for alizarin red staining for mineralization (Figure 1B).

**FIGURE 2:**
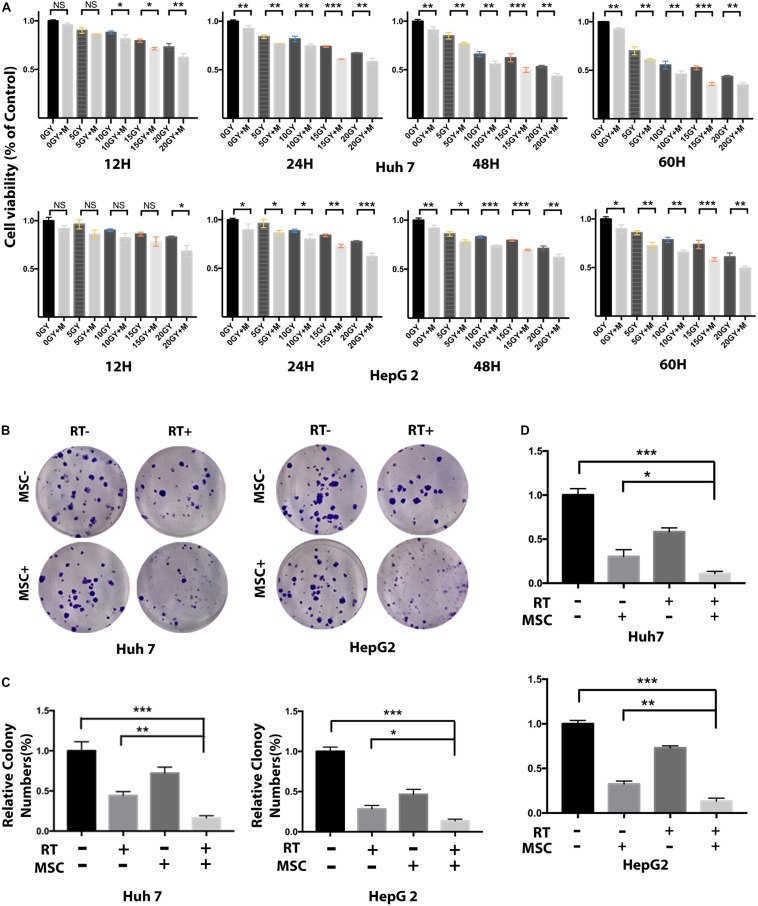
AT-MSC augments the effects of RT against HCC cells proliferation and sphere-forming. **(A)** Cell viability of Huh7 and HepG2 cells treated with radiation and/or AT-CM for 12, 24, 48, and 60 h was assessed by CCK8 assay. Data represent the means ± SD of three independent experiments (^∗∗^*P* < 0.01, ^∗∗∗^*P* < 0.001). **(B,C)** Huh7 cells treated with RT with or without AT-CM were cultured on ultra-low attachment plates to assess sphere-formation ability. Representative images are shown. The results are expressed as means ± SD of three independent experiments (^∗^*P* < 0.05, ^∗∗^*P* < 0.01, ^∗∗∗^*P* < 0.001). **(D)** The effect of AT-MSCs co-culture with human HCC cell line Huh7 and HepG2 after irradiation. Tumor cells grown as colonies in a monolayer culture. The initial size of the colonies was measured and successive treatments was applies for 7 days more.

We co-cultured AT-MSCs with Huh7 and HepG2 cell colonies to address the combination effect on tumor growth. After treatments, the areas occupied with the colonies were measured on three consecutive days. Quantification of the total colony area showed a significant growth delay in the RTM group, compared to the same colony grown in CTRL, RT, and MSC group (Figure 2D).

**FIGURE 3:**
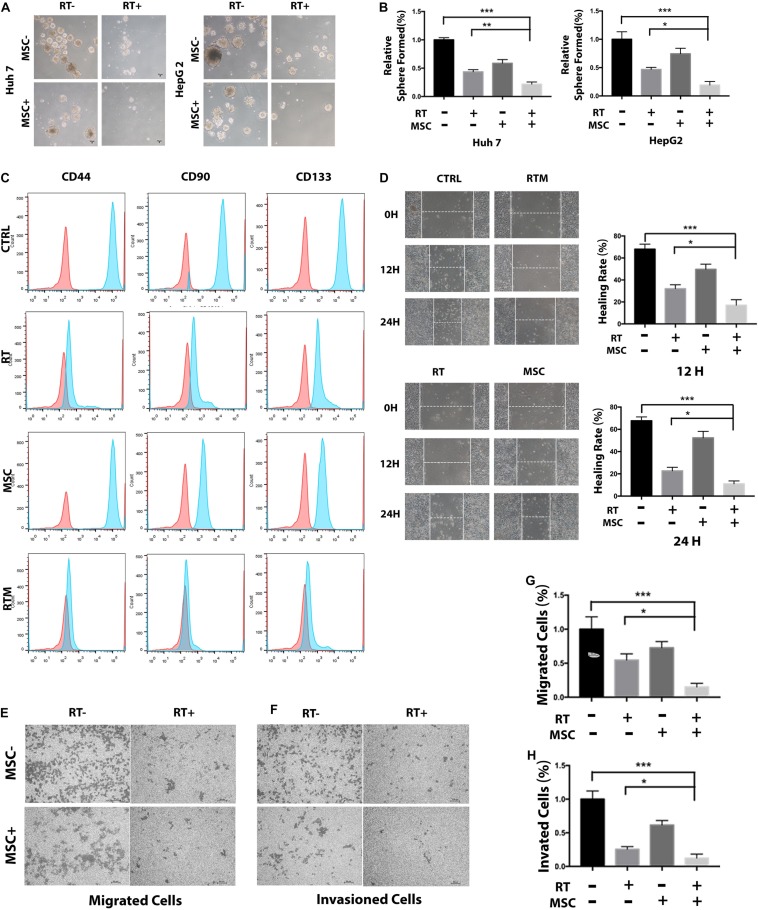
AT-CM in combination with RT against HCC cells colony-forming, wound-healing, migration, and invasion. **(A,B)** The colony-formation assay showed decreased tumorigenicity in Huh7 cells, which treated with RT and AT-CM combination therapy. The graph shows the mean integrated density of colonies normalized by 100%. **(C)** Tumor cells exposed to irradiation with or without AT-CM were fixed and then CD44, CD90, and CD133-positive cells were analyzed with FACS. Data are from representative experiments repeated at least three times. **(D)** Representative wound healing assays (12 and 24 h) after scratching (0 h) demonstrated that the combination treatment reduced the wound closure ability of Huh7 cells. Representative images are shown (magnification 100 ×). **(E–H)** Transwell migration and invasion assays were performed to compare the motility of HCC cells treated with RT with or without AT-CM. The numbers of migrated (top) and invading (bottom) cells were calculated and are depicted in the bar chart (^∗^*P* < 0.05, ^∗∗^*P* < 0.01, ^∗∗∗^*P* < 0.001).

Next, we evaluated the effects of AT-CM and RT combination treatment on cell cytotoxicity, proliferation, sphere formation and colony formation in HCC cell lines Huh7 and HepG2. Cell viability was assessed after 12, 24, 48, and 60 h of exposure to different doses of radiation, AT-CM and non-conditioned control medium. The CCK8 results showed that AT-CM combined with RT significantly decreased the viability of HCC cells (Figure 2A). As expected, both sphere formation (Figures 2B,C) and colony formation (Figures 3A,B) were impaired in the RTM group compared with CTRL, RT, and MSC group. After the treatments, we investigated the effects of RT combined AT-MSCs treatment on the wound healing, migration and invasion of the tumor cells. The wound healing (Figure 3D), migration and invasion abilities (Figures 3E–H) of HepG2 and Huh7 cells were significantly inhibited in RTM group. Furthermore, several surface markers such as CD90, CD44, CD133 were analyzed using flow cytometry to measure the cancer stem cell population (Figure 3C). The combination treatment of RT and AT-CM decreased the expression of CD44, CD90, and CD133 in Huh7 cells.

### AT-MSCs Enhances the Anti-HCC Effect of RT *in vivo*

**FIGURE 4:**
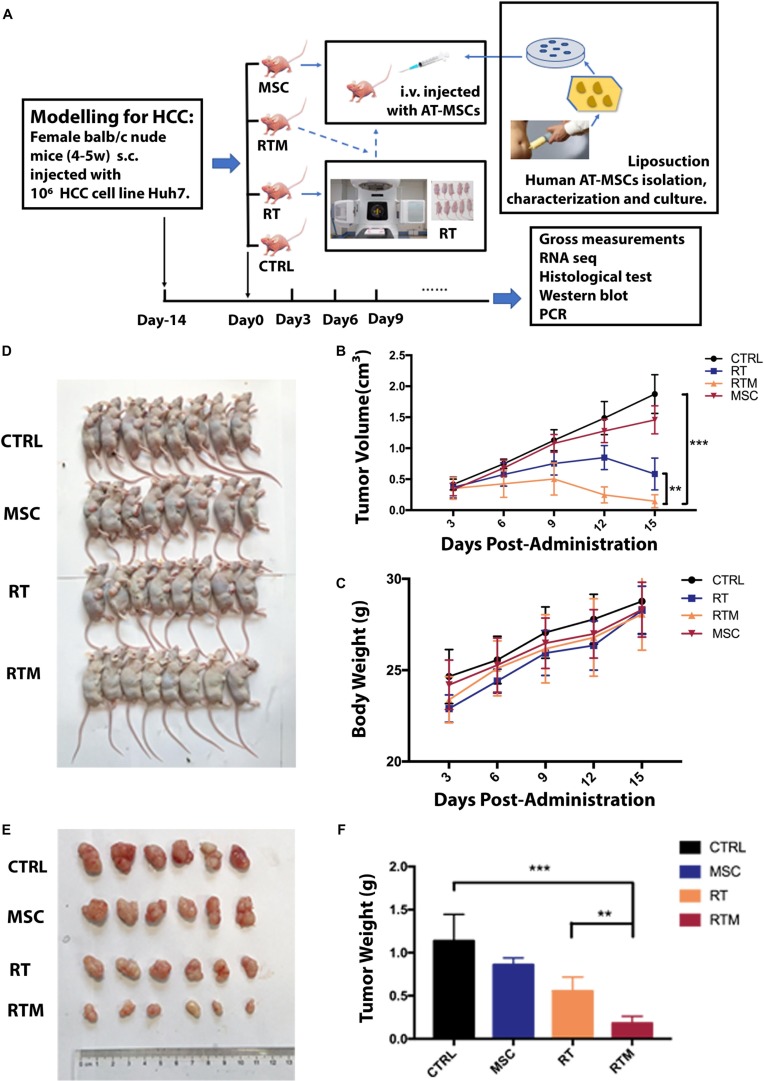
Adipose tissue-derived mesenchymal stem cells (AT-MSCs) enhance the anti-HCC effects of RT efficiently *in vivo*. **(A)** Schematic illustration of animal study schedule. Flowchart showing the preparation and culture of AT-MSCs. Treatment was started when the tumor reached ∼100 mm^3^, designed as day 0. Orthotopic xenografts of HCC cells in female Balb/c nude mice were randomly divided into four groups: (1) CTRL group: tumors were injected with saline; (2) RT group: tumors were treated with radiation; (3) MSC group: tumors were treated with administrated 1 × 10^6^ AT-MSCs; and (4) RTM group: tumors were injected with AT-MSCs via the tail vein on day 0 immediately after radiation exposure. **(B)** Subcutaneous tumor volumes (mm^3^) in all groups was plotted. Data represent the mean tumor volume in each group measured on the indicated day (*n* = 10 mice per group. ^∗∗^*P* < 0.01, ^∗∗∗^*P* < 0.001). **(C)** Body weight of mice after treatment at the indicated time points (*n* = 10 mice per group). **(D)** Representative images of mice with induced tumors at the endpoint. **(E)** Tumors from each group were excised on the indicated day. **(F)** The net tumor weight of mice in each treatment group. Tumors were harvested, and the weights were measured before lysis at the endpoint (*n* = 8 tumors per group. ^∗∗^*P* < 0.0, ^∗∗∗^*P* < 0.001).

**FIGURE 5:**
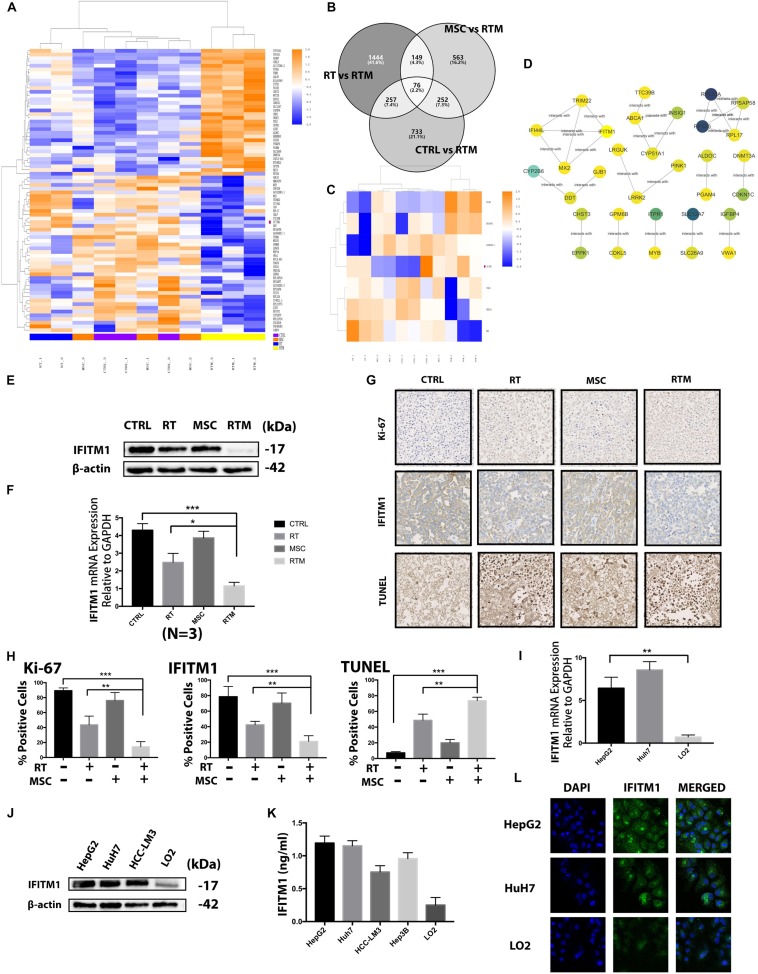
Screening of the functional gene in the combination strategy against HCC. **(A)** Heatmap of the RNA sequencing results. Changes in the relative abundance of proteins and components from Huh7 tumor-bearing mice treated with RT with or without AT-MSCs (*n* = 2–3 per group). **(B–C)** Venn diagram and heatmap showing 76 genes differentially expressed between the CTRL and RTM, RT and RTM, and MSC and RTM groups. **(D)** Functional interaction sub-network constructed using module genes. **(E)** Western blot analysis demonstrating IFITM1 expression in HCC xenograft tumor samples. β-Actin was used as a loading control. **(F)** Detection of IFITM1 mRNA expression in xenograft tumor samples by qRT-PCR after RT with or without AT-MSCs treatment. Fold change expression was calculated with normalization to GAPDH as the internal control gene. Each value represents the mean ± SD from three independent experiments (^∗^*P* < 0.05, ^∗∗∗^*P* < 0.001). **(G)** Representative images of IHC staining of the proliferative marker Ki-67 (top) and IFITM1 and TUNEL staining (bottom) in xenograft tumors (*n* = 3–5 tumors per group, five images per tumor. Scale bar = 100 μm). **(H)** The percentages of Ki- 67-, IFITM1-, and TUNEL-positive cells are summarized in the bar chart (^∗∗^*P* < 0.01, ^∗∗∗^*P* < 0.001). **(I)** Protein expression of IFITM1 in HCC cells and the immortalized liver cell lines LO2 was detected by Western blot analysis. β-actin was used as a loading control. **(J)** Detection of IFITM1 mRNA expression in HCC cell lines and LO2 by qRT-PCR. The results are expressed as the means ± SD of three independent experiments (^∗∗^*P* < 0.01). **(K)** Detection of secreted IFITM1 level in HCC cell lines and immortalized liver cell lines. **(L)** Representative image of immunofluorescence staining of IFITM1 (green) in indicated cells; nuclei are stained with DAPI (blue).

To determine whether AT-MSCs synergize with RT *in vivo*, a preclinical Huh7 xenograft model was validated. To simulate the clinical procedure for patients, mice were sorted into four groups (Figure 4A). As expected, the RTM combination regimen was the most effective, resulting in a significantly lower mean tumor volume and mean tumor weight at the endpoint (Figures 4B,D–F). No weight loss was observed in the *in vivo* experiment (Figure 4C). To evaluate cell proliferation in the xenograft tumors, IHC staining of the proliferative Ki67 marker was conducted; tumors receiving the RT and AT-MSCs combination treatment showed a significantly lower percentage of positive Ki67 staining, indicating the tumor-suppressive function of AT-MSCs (Figure 5E, top). To evaluate apoptosis in the tumor lesions, a TUNEL assay was performed in tumor samples obtained from each mouse. The results showed that metastatic lesions from the RTM group had a significantly higher apoptotic index compared with those from the CTRL group (Figure 5E, bottom).

### Results of RNA-seq Analysis

**FIGURE 6:**
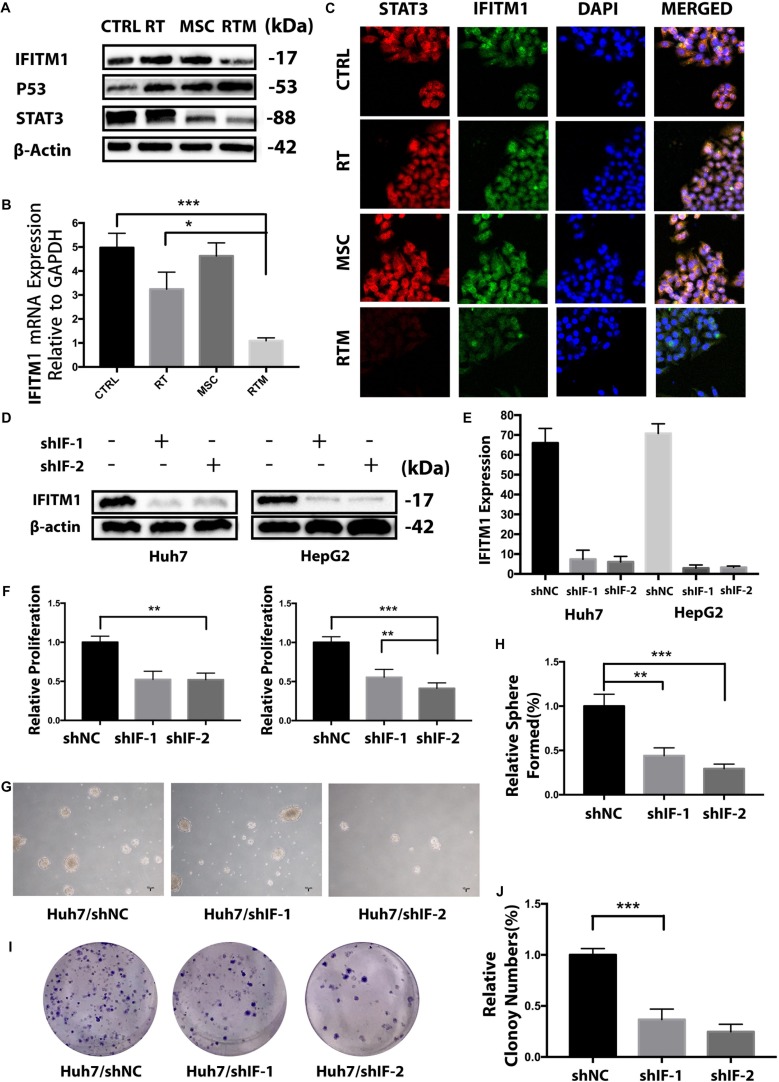
IFITM1 is identified as a potential HCC-oncogene and involves in the synergy treatment. **(A,B)** Detection of IFITM1 protein and mRNA expression in Huh7 cells treated with RT with or without AT-MSCs by Western blot analysis and qRT-PCR. β-Actin was used as a loading control. **(C)** Representative image of immunofluorescence staining of STAT3 (red) and IFITM1 (green) in the indicated cells; nuclei are stained with DAPI (blue). **(D,E)** HCC cell lines were transduced with shRNAs targeting IFITM1, and the expression level of IFITM1 was assessed by Western blot analysis and qRT-PCR using β-Actin and GAPDH as loading controls, respectively. The results are expressed as means ± SD of three independent experiments. **(F)** The viability of Huh7 and HepG2 cells transfected with shRNAs were assessed by CCK8 assay for 24 h. Data represent the means ± SD of three independent experiments (^∗∗^*P* < 0.01, ^∗∗∗^*P* < 0.001). **(G,H)** Huh7 cells transfected with shRNAs targeting IFITM1 (shIF-1 or shIF-2) or negative control (NC) shRNA were cultured on ultra-low attachment plates to determine the sphere-formation ability. Representative images are shown. The results are expressed as the means ± SD of three independent experiments (^∗^*P* < 0.05, ^∗∗^*P* < 0.01, ^∗∗∗^*P* < 0.001). **(I,J)** A colony-formation assay showed decreased tumorigenicity of Huh7 cells transfected with shIF-1 or shIF-2 compared with NC shRNA *in vitro*. The graph shows the mean integrated density of colonies normalized by 100% (^∗∗∗^*P* < 0.001).

Next, we elucidated the mechanisms underlying the synergistic effects of AT-MSCs and RT combination treatment in the four experimental groups using RNA sequencing and high-throughput analysis to identify markers. With the criteria of a fold change ≥2 and adjusted-*P* < 0.05, 76 genes were found in comparisons of the RTM vs. CTRL, RTM vs. RT, and RTM vs. MSC groups (Figures 5A–C). Correlations among the genes were calculated using ReactomeFIViz network-based functional analysis tool. Markov cluster algorithm (MCL) was applied to generate modules, by applying the size ≥3 as a cutoff to filter out network (Figure 5D) To evaluate which of these RNAs may be related to the synergistic effects of the combination treatment, we performed a validation analysis using the NCBI Gene database to determine whether the sequence matched to that of a human RNA sequence, and also conducted a comprehensive literature search of the genes in relation to carcinogenesis to identify the biological functions of each gene. Based on the average log2 ratio values and previous studies (Gao et al., 2015; Lui et al., 2017; Yang J. et al., 2018), the RNA IFITM1 gene, which was downregulated in RTM group, was selected for analysis in further experiments after confirmed *in vitro* experiment (Figure 6B). We performed Western blot, qRT-PCR and IHC analyses on mouse tumors, and found decreased IFITM1 expression in RTM group compared to other groups (Figures 5E–H). We hypothesized that the RT and AT-MSCs combination therapy augment the efficacies of each individual treatment by downregulating IFITM1 expression in HCC cell lines.

### Identification of IFITM1 as a Tumorigenesis Related Genes

The mRNA and protein levels of IFITM1 in human HCC cell lines were analyzed by qRT-PCR, Western blotting and immunofluorescence analysis, respectively (Figures 5I,J,L). IFITM1 was highly expressed in almost all HCC cell lines and the protein levels were consistent with the mRNA levels. To evaluate the secreted protein, CM of those cell lines were collected and was performed with ELISA assay. Consistently, the level of secreted IFITM1 in HCC cell lines was higher than immortalized liver cell line LO2 (Figure 5K).

To elucidate the functional role of IFITM1 in HCC cell carcinogenesis, we examined the effect of IFITM1 mRNA knockdown on HCC cell growth by transfecting HCC cells with shRNA specific to IFITM1 or non-sense control shRNA (shNC). Both shRNAs dramatically decreased the IFITM1 expression level compared with vector transfected (Figures 6D,E).

To assess the role of IFITM1 in HCC cell lines growth, we evaluated the proliferation of IFITM1-depleted cells using the CCK8 assay. Transfection with IFITM1-specific shRNA, but not shNC, statistically reduced the growth of Huh7 and HepG2 cells (Figure 6F), suggesting that cell proliferation was negatively regulated in the absence of IFITM1. We performed a sphere-formation assay to determine whether IFITM1 contributed to the capacity of cancer cells to proliferate and self-renew. Results showed that IFITM1 knockdown derivatived the sphere formation ability of HCC cell lines which were increasingly decreased starting from secondary plating, reaching a significant difference (Figures 6G,H). Similarly, the *in vitro* quantitative colony-formation assay also showed less colony growth after IFITM1 silencing (Figures 6I,J). Collectively, these functional experiments indicate that the tumorigenic properties of hepatoma might be correlated with IFITM1 expression.

### IFITM1 Interacts With STAT3 and Matrix Metallopeptidases (MMPs) and Inhibits Downstream p53 and Caspases

As IFNγ signaling has been reported to interact with STAT3, which is considered to promote many tumors including HCC, we next studied whether IFITM1-mediated effects in RT and AT-MSCs combination therapy could affect the expression level of STAT3 (He et al., 2010; Wang et al., 2011).

**FIGURE 7:**
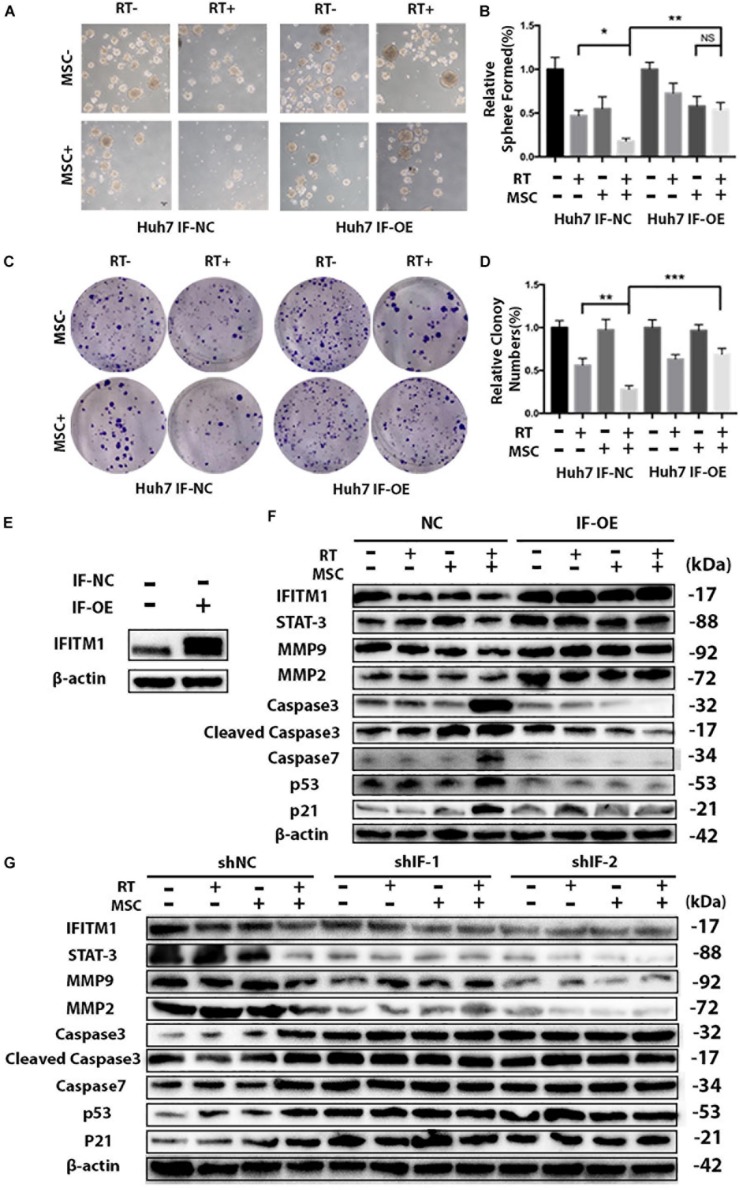
IFITM1 interacts with STAT3 and MMPs and inhibits downstream p53 and Caspases. **(A)** Control (IF-NC) and IFITM1-overexpression (IF-OE) Huh7 cells treated with RT with or without AT-CM were cultured on ultra-low attachment plates to assess sphere-formation ability. Representative images are shown. **(B)** The results are expressed as means ± SD of three independent experiments (^∗^*P* < 0.05, ^∗∗^*P* < 0.01). **(C)** The colony-formation assay showed increased tumorigenicity in IF-OE Huh7 cells. **(D)** The graph shows the mean integrated density of colonies normalized by 100% (^∗∗^*P* < 0.01, ^∗∗∗^*P* < 0.001). **(E)** We overexpressed IFITM1 in Huh7 cells using lentivirus-mediated transcription, and the expression level of IFITM1 was assessed by Western blot analysis using β-Actin as loading control. **(F)** IF-NC and IF-OE Huh7 cells treated with RT with or without AT-CM and cell lysates were isolated to probe with antibodies indicated. Protein level was determined by western blot with β-actin used as the loading control. Representative images were shown. **(G)** Cell lysates were isolated from HCC Huh7 cells after knocking down IFITM1, and the protein levels were probed with antibodies indicated. β-actin used as the loading control. Representative images were shown.

Immunofluorescence data showed that IFITM1 co-localized with STAT3 in the combination therapy (Figure 6C), and western blot results showed that IFITM1 influenced the level of STAT3 in the combination therapy (Figure 6A). Furthermore, silencing of IFITM1 in Huh cells significantly repressed STAT-3 in the RT and AT-MSCs combination group (Figure 7G), which was consistent with previous studies (Eferl, 2012). Also, we found that loss of IFITM1 markedly induced p53 and p21 transcription, which play important roles in cell survival (Figure 7G). The typical molecular indicators of apoptosis, cleaved-caspase3, caspase 3, and caspase 7 were also found increased more rapidly in IFITM1-silencing cells (Figure 7G). Epithelial-mesenchymal transition (EMT) markers such as MMP2 and MMP9 were downregulated in RT and AT-MSCs combination therapy, whereas the expressions were inhibited in the absence of IFITM1 (Figure 7G).

### Overexpression of IFITM1 Reverses the Effects of Combination Therapy of AT-CM and RT on HCC Cell Line

To validate the importance of IFITM1 in the combination therapy of RT and AT-MSCs, we overexpressed IFITM1 in Huh7 cells using lentivirus-mediated transcription. Functional assays revealed that IFITM1 overexpression could increase the efficiency of sphere formation and colony formation in RTM combination therapy group (Figures 7A–D), compared with NC-transfected cells.

To real the mechanisms by which IFITM1 overexpression led to the impaired ability of cancer stem cell-like properties and EMT signaling in HCC cell lines, protein was isolated and analyzed with Western blot. Notably, we found that overexpression of IFITM1 was associated with increased STAT3 and MMPs expressions, and significantly reduced caspases, p53 and p21 (Figures 7E,F).

## Discussion

With advancements in modern radiotherapy and imaging techniques, RT technologies have allowed delivery of ablative doses to HCC without affecting normal liver tissue, thus creating a shift from palliative to cure-oriented treatment options (Ma et al., 2010). However, most patients have advanced HCC at the time of diagnosis and are at high risk of distant failure. Interest in using combined systematic therapy with RT to maximize outcomes is growing (Keane et al., 2018). With the development of targeted therapies, the numbers of studies investigating the potential of combination therapy involving radiation is increasing. The use of sorafenib as first-line systematic therapy for patients with advanced HCC and poor outcomes is of great interest (Rimassa and Santoro, 2009). Although preclinical evidence suggested that sorafenib is a radiosensitizer and potential therapy against HCC, it has been challenging to demonstrate this in the clinic, largely because of a significant potential for increased toxicity (Brade et al., 2016). Meanwhile, synergy between RT and immune checkpoint blockade (ICB) is an intriguing area of research. RT regulates immunogenic modulation in the tumor microenvironment, including accumulation of cancer cell surface molecules and tumor-associated antigens, and therefore might promote tumor susceptibility to ICB (Demaria and Formenti, 2013; Sharabi et al., 2015). However, much of the existing evidence is for melanoma, whereas that for combination therapy for HCC is very limited (Keane et al., 2018), likely due to the unsatisfactory results of ICB alone in HCC (Friedman et al., 2017).

Adult stem cells are located in essentially all tissues and organs of the human body, including the skin, skeletal muscle, blood vessels, liver, intestine, bone marrow, and adipose tissue (Bacakova et al., 2018). Due to their tumor-homing properties, as well as the inherent anticancer potential and potential to deliver anti-tumor agents directly to the tumor site, MSCs are gaining attention for use in clinical applications (Studeny et al., 2002; Ciavarella et al., 2012; Ho et al., 2013). Human MSCs (hMSCs) decreased the malignant phenotypes of human hepatoma cell lines (H7402 and HepG2) in an animal transplantation model (co-culture systems and CM from hMSCs) both *in vivo* and *in vitro* (Qiao et al., 2008b). Western blot analysis and immunofluorescence staining provided evidence that Wnt signaling pathway has a role in MSCs-induced tumor inhibition (Neth et al., 2006). This suggests that components involved in MSC proliferation and differentiation might be subverted into molecular mechanisms that underlie malignant. Previous studies demonstrated that genetically engineered MSCs with anticancer activity is also shown as a promising approach in exerting therapeutic effects. For instance, Mohammadpour et al. used recombinant TNF-α-activated MSCs in combination with RT in breast cancer model; Sasportas et al. shown the possibility of engineering hMSCs in 2 different mouse models of glioma, demonstrating potential clinical value in cancer treatment (Sasportas et al., 2009; Mohammadpour et al., 2016). In comparison with other MSCs pools, AT-MSCs are much more accessible due to their subcutaneous location and abundance (Bacakova et al., 2018). Of note, AT-MSCs have been used at the Mayo Clinic for all subjects enrolled in the previous study, and a phase III randomized, double-blind, controlled, clinical trial showed that allogeneic adipose-derived stem cells (Cx601) are both effective and safe for patients with Crohn’s disease with complex perianal fistulas (Panes et al., 2016). A systematic review focusing on the safety of adipose-derived cell therapy included five studies of patients with previous malignancies, only 1 of the 121 total patients in those studies was reported to develop breast cancer recurrence. These results suggest that At-MSCs therapy has a favorable safety profile.

The present study showed for the first time that AT-MSCs might act synergistically with RT. Using a cell-derived xenograft model of HCC cells, RT treatment followed by AT-MSCs administration augmented the anti-cancer efficacy, with a longer latency and smaller mean tumor volume. As there is no previous research on this type of combination therapy, we used a time window recommended by clinical studies of combination radioimmunotherapy (Formenti et al., 2018; Roger et al., 2018). The following therapies are ranked from highest to lowest efficacy *in vivo:* HDR combined with AT-MSCs, HDR, low-dose radiation (LDR) and LDR combined AT-MSCs. Thus, we chose 15 Gy in subsequent experiments *in vitro*. Similar to the previous findings that in contrast to LDR, HDR triggered more tumor antigens, rendering cancer cells more sensitive to T-cell attack (Reits et al., 2006). We provide evidence that synergy between RT and AT-MSCs promotes apoptosis and suppresses proliferation, migration, and invasion via inhibition of oncogenic IFITM1 expression in HCC cells. The work of Robert Eferl had summed up the functions of IFNγ signaling in hepatic injury, inflammation, aging and HCC (Eferl, 2012). It is indicated in the figure that IFNγ is compromised and STAT3 enhanced. We assumed that there might exist new upstream or other suppressing factors of IFIM1 in this combination therapy to causing the downregulation of IFITM1.

Emerging research suggests that IFN signaling plays an important role in the tumor microenvironment and affects anti-cancer therapies differentially (Diamond and Farzan, 2013; Liu et al., 2018; Patin et al., 2018). IFITM1 is a member of the IFN-stimulated gene (ISG) family and an essential mediator of interferon-alpha, -beta and -gamma-mediated inhibition of cell proliferation and cell adhesion signaling (Johnson et al., 2006; Fu et al., 2017). It is largely known for the importance in many cellular functions, such as proliferation and adhesion. Much evidence has shown that IFITM1 is essential for tumorigenesis, and its overexpression is positively correlated with tumor progression and invasiveness (Lui et al., 2017; Yang Y.G. et al., 2018). Recently, increasing studies have shown overexpression of IFITM1 in a variety of cancers, such lung, gastrointestinal and colorectal cancers (Andreu et al., 2006; Lee et al., 2012; Yan et al., 2019). However, interactions between cancer and IFITM1 are controversial. It has been discovered that IFITM1 could act a tumor suppressor role in HCC (Yang et al., 2007). This study use hepatoma line BEL-7404 and QSG-7701, and we use Huh7 and HepG2 cell lines. In our opinion, different cell lines might have diverse response to a same gene modification. That’s why there is no unanimous conclusion about the role of IFITM1 in HCC. A global analysis of DNA methylation identified IFITM1 as a candidate hypermethylated gene (Gao et al., 2015). An inverse correlation between IFITM1 and p53 expression was also found in human HCC cell lines under matrine treatment (Xie et al., 2015), same relationship with p21 was found in breast cancer (Lui et al., 2017). STAT3 is considered to promote formation of many tumors including HCC, and the mutual interplay between STAT3, caspases and MMPs and IFITM1 was revealed by Meng et al. (Eferl, 2012). More recently, IFITM1 was identified as a radioresistance gene by analysis of the GEO database and verified by diverse experiments (Yang J. et al., 2018). It has been suggested that the combination of IFITM1 knockdown with RT inhibited oral neoplasms.

**FIGURE 8:**
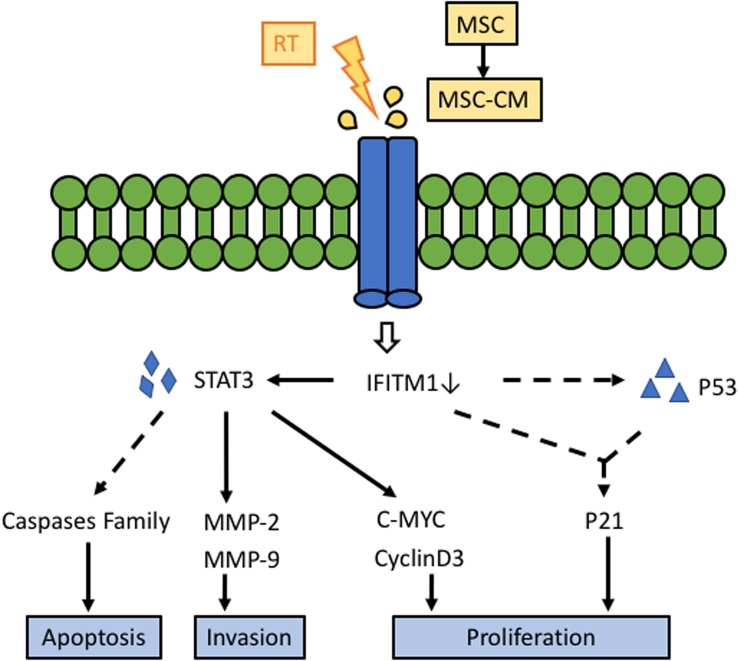
Schematic diagram of the potential effect of the RT and AT-MSCs combination therapy on HCC. Our results suggest that the synergistic treatment effect of RT and AT-MSCs has therapeutic potential for HCC in mice via the inhibition of IFITM1 expression, inhibition of HCC cell proliferation via upregulation of p53 and p21, inhibition of tumor invasion via downregulation of MMPs, and promotion of HCC cell apoptosis. AT-MSCs: adipose tissue-derived mesenchymal stem cells; AT-CM: conditioned medium (CM) from AT-MSCs; IFITM1: interferon-induced transmembrane protein 1; STAT3: signal transducer and activator of transcription 3; MMP: matrix metallopeptidases.

Although we have discovered that the combination of RT and AT-MSCs could inhibit HCC cells through regulation of IFITM1 and its downstream (Figure 8), specific molecular targets and correlated pathways are still needed to be explored further. We acknowledge limitations to our study. MSCs differentiation and paracrine signaling act in the local microenvironment either separately or together simultaneously. We treated the xenograft model with AT-MSCs, but in the *in vitro* experiment, we exposed HCC cell lines to AT-MSC-CM, thus eliminating any direct cell-to-cell effects. Although the safety concerns of MSCs-induced tumorigenicity has decreased through the exploration of cell-based therapies, it is important to assess MSCs differentiation in the various lineages involved in the procedure. In addition, time-course studies analyzing the most appropriate time window for the combination of RT and AT-MSCs are needed.

In conclusion, we showed for the first time that AT-MSCs enhanced the efficacy of radiotherapy in HCC cell *in vitro* by silencing IFITM1 expression and significant delaying the growth of HCC tumors *in vivo*.

## Data Availability Statement

The raw data supporting the conclusions of this manuscript will be made available by the authors, without undue reservation, to any qualified researcher.

## Ethics Statement

The studies involving human participants were reviewed and approved by The First Affiliated Hospital, Zhejiang University School of Medicine. The patients/participants provided their written informed consent to participate in this study. The animal study was reviewed and approved by The First Affiliated Hospital, Zhejiang University School of Medicine. Written informed consent was obtained from the individual(s) for the publication of any potentially identifiable images or data included in this article.

## Author Contributions

LW wrote the manuscript and participated in performing the experiments and analyzing the data. QT and XY took part in performing the experiments and analyzing the data. DY wrote the revised manuscript and participated in performing the experiments. MT investigated the project. JX and QP curated data and took part in formal analysis. CM conceived and designed the experiments and contributed to the reagents, materials, and analysis tool. SY conceived and designed the experiments and made the final approval of the version to be published.

## Conflict of Interest

The authors declare that the research was conducted in the absence of any commercial or financial relationships that could be construed as a potential conflict of interest.
