# Supplementary material for: The Therapeutic Potential of Adipose Tissue-Derived Mesenchymal Stem Cells to Enhance Radiotherapy Effects on Hepatocellular Carcinoma
Source: Front Cell Dev Biol. 2019 Nov 12;7:267. doi: 10.3389/fcell.2019.00267 (PMC6861425; doi:10.3389/fcell.2019.00267)
Supplement: Supplementary file 2 [file Data_Sheet_2.PDF]

Cell Line Authentication Service
STR Profile Report

**Sample Submitted By:**	Dr. Weilin Wang<br>Zhejiang University
**Email Address:**	wam@zju.edu.cn
**Sales Order:**	180905J
**Cell Line Designation:**	HuH-7
**Date Sample Received:**	Sep 5th, 2018
**Report Date:**	Sep 6th, 2018

Methodology: Nineteen short tandem repeat (STR) loci plus the gender determining locus, Amelogenin, were amplified using the commercially available EX20 Kit from AGCU. The cell line sample was processed using the ABI Prism® 3130 XL Genetic Analyzer. Data were analyzed using GeneMapper® ID v3.2 software (Applied Biosystems). Appropriate positive and negative controls were run and confirmed for each sample submitted.

Data Interpretation: Cell lines were authenticated using Short Tandem Repeat (STR) analysis as described in 2012 in ANSI Standard (ASN-0002) by the ATCC Standards Development Organization (SDO) and in Capes-Davis et al., Match criteria for human cell line authentication: Where do we draw the line? Int J Cancer. 2013;132(11):2510-9.

GTB™ performs STR Profiling following ISO 9001:2008 and ISO/IEC 17025:2005 quality standards.
There are no warranties with respect to the services or results supplied, express or implied, including, without limitation, any implied warranty of merchantability or fitness for a particular purpose. Genetic Testing Biotechnology (GTB) is not liable for any damages or injuries resulting from receipt and/or improper, inappropriate, negligent or other wrongful use of the test results supplied, and/or from misidentification, misrepresentation, or lack of accuracy of those results. Your exclusive remedy against GTB and those supplying materials used in the services for any losses or damage of any kind whatsoever, whether in contract, tort, or otherwise, shall be, at GTB's option, refund of the fee paid for such service or repeat of the service.

The GTB™ is a registered trademark of Genetic Testing Biotechnology Corporation (Suzhou).

Technical Questions?
GTB Technical Support
+86-512-67486171
service@jsdna.org
Section 505, Yixin BLD
SIP, Suzhou, 215123
Jiangsu, P.R. China

Ordering Questions?
order@jsdna.org
GTB Corporation
+86-512-62806339
Section 303, Yixin BLD
SIP, Suzhou, 215123
Jiangsu, P.R. China

GENETIC TESTING BIOTECHNOLOGY

Cell Line Authentication Service
STR Profile Report

Sales Order: 180905J

	Test Results for Submitted Sample		DSMZ Reference Database Profile	
Loci	Query Profile: HuH-7		Database Profile: HuH-7	
Amelogenin	X		X	
D3S1358	15			
D13S317	10	11	10	11
D7S820	11		11	
D16S539	10		10	
Penta E	11			
TPOX	8	11	8	11
TH01	7		7	
D2S1338	19			
CSF1PO	11		11	
Penta D	12			
D19S433	13	14		
vWA	16	18	16	18
D21S11	30			
D18S51	15			
D6S1043	13	15		
D8S1179	14	15		
D5S818	12		12	
D12S391	20	21		
FGA	22			

The allele match algorithm compares the 8 core loci plus amelogenin only, even though alleles from all loci will be reported when available.

*Note: Loci highlighted in grey (8 core STR loci plus Amelogenin) can be made public to verify cell identity. In order to protect the identity of the donor, **please do not publish** the allele calls from all the STR loci tested.*

Explanation of Test Results

Cell lines with ≥80% match are considered to be related; i.e., derived from a common ancestry. Cell lines with between a 55% to 80% match require further profiling for authentication of relatedness.

- ☐ The submitted sample profile is human, but not a match for any profile in the DSMZ STR database.
- ☑ The submitted profile is an exact match for the following human cell line(s) in the DSMZ STR database (8 core loci plus Amelogenin): HuH-7
- ☐ The submitted profile is similar to the following DSMZ human cell line(s):

e-Signature, Technician:

Digitally signed by Faye Wong
DN: cn=Faye Wong, o=Genetic Testing Biotechnology (Suzhou), ou=DNA Typing Section, email=order@jsdna.org, c=CN
Date: 2018.09.06 18:28:29 +08'00'

GENETIC TESTING BIOTECHNOLOGY
GUARANTEE
CELL LINE AUTHENTICATION

e-Signature, Reviewer:

Digitally signed by Alan Cui
DN: cn=Alan Cui, o=Genetic Testing Biotechnology (Suzhou), ou=Supervision Section, email=service@jsdna.org, c=CN
Date: 2018.09.06 18:31:14 +08'00'

More information

Addendum: Electropherogram for the customer's sample set 1 of 1

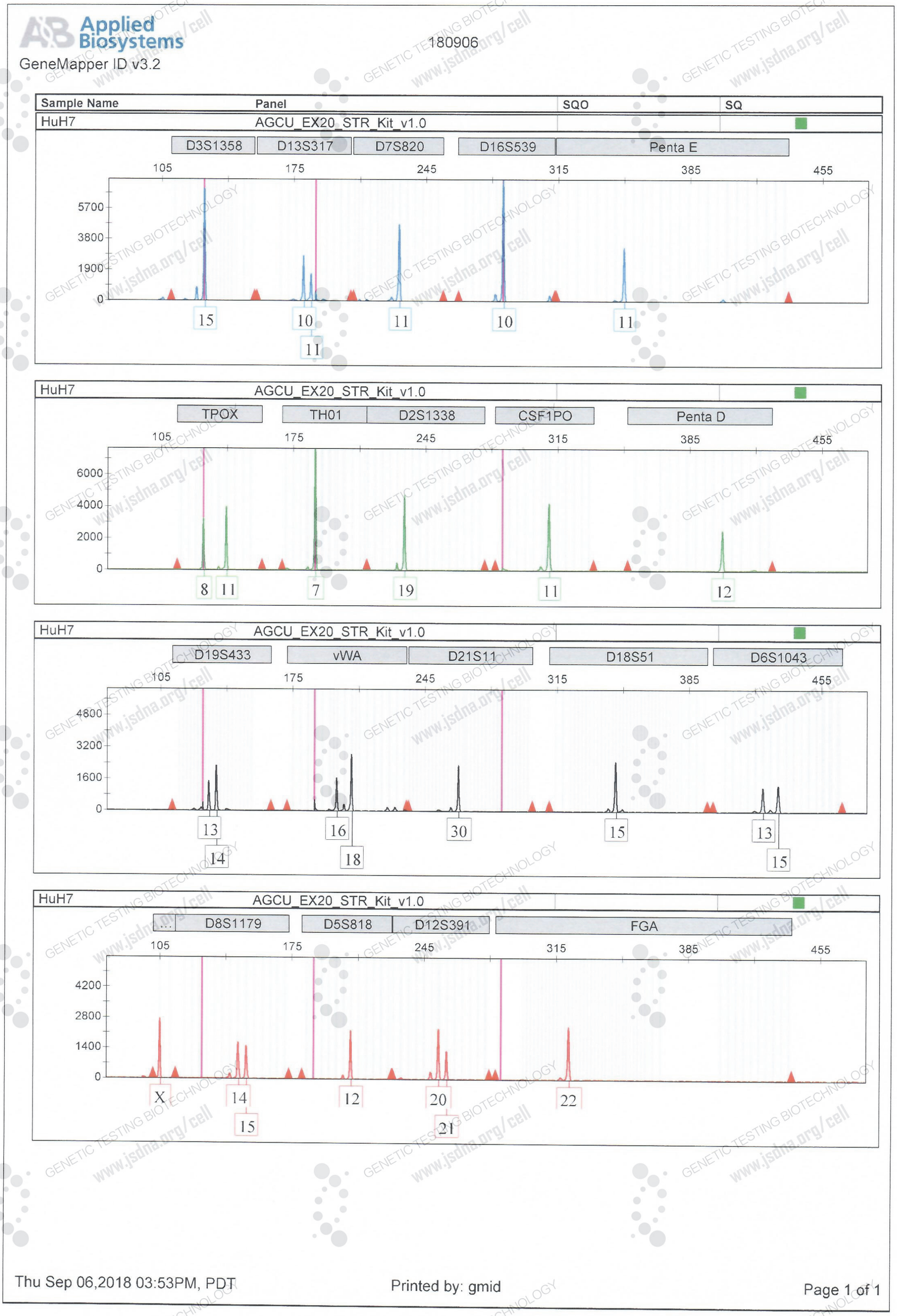
Applied Biosystems
GeneMapper ID v3.2
180906
Sample Name
Panel
SQO
SQ
HuH7
AGCU_EX20_STR_Kit_v1.0
D3S1358
D13S317
D7S820
D16S539
Penta E
15
10
11
11
10
11
TPOX
TH01
D2S1338
CSF1PO
Penta D
8
11
7
19
11
12
D19S433
vWA
D21S11
D18S51
D6S1043
13
14
16
18
30
15
13
15
D8S1179
D5S818
D12S391
FGA
X
14
15
12
20
21
22
Thu Sep 06,2018 03:53PM, PDT
Printed by: gmid
Page 1 of 1
