# Supplementary material for: The Therapeutic Potential of Adipose Tissue-Derived Mesenchymal Stem Cells to Enhance Radiotherapy Effects on Hepatocellular Carcinoma
Source: Front Cell Dev Biol. 2019 Nov 12;7:267. doi: 10.3389/fcell.2019.00267 (PMC6861425; doi:10.3389/fcell.2019.00267)
Supplement: Supplementary file 3 [file Table_1.docx]

**Table S1. Materials and reagents.**

| Names | Company | Category No. |
| --- | --- | --- |
| Adipogenic differentiation medium | Cyagen Biosciences, Guangzhou, CHN | HUXMA-90031 |
| B-27 | Gibco, Grand Island, NY, USA | 17504044 |
| bFGF | Gibco, Grand Island, NY, USA | 13256029 |
| Cell Counting kit-8 | Dojindo, Kumamoto, Japan | EX783 |
| DAB Substrate Kit | Abcam, Cambridge, MA, UK | ab64238 |
| Dulbecco’s modified Eagle’s medium (DMEM) | Invitrogen Corporation, Carlsbad, CA, USA | 11995065 |
| DMEM/F-12 | Gibco, Grand Island, NY, USA | 11330057 |
| Dimethylsulfoxide (DMSO) | Sigma Chemical Corporation, St.Louis, MO, USA | D4540 |
| EGF | Gibco, Grand Island, NY, USA | PHG0311 |
| Fetal bovine serum (FBS) | Invitrogen Corporation, Carlsbad, CA, USA | 10099-141 |
| Osteogenic differentiation medium | Cyagen Biosciences, Guangzhou, CHN | HUXMA-90021 |
| QuantiTect Reverse Transcription Kit | Translational Medicine, Suzhou, CHN | 205311 |
| SYBR Green1 | TAKARA,Dalian, CHN | RP420A |
| SYBR Premix Ex TaqTM(200T) | TAKARA,Dalian, CHN | RR420A |
| SuperSignal^TM^ West Femto Maximum Sensitivity Substrate | Thermo Fisher, Waltham, USA | 34096 |
| Trizol | Invitrogen Corporation, Carlsbad, CA, USA | 15596-026 |
| Trypsin | Promega, Madison, WI, USA | V5117 |
| Trypsin/EDTA | Invitrogen Corporation, Carlsbad, CA, USA | 25200-072 |
| TUNEL staining | Roche Diagnostics GmbH, Mannheim, Germany | 12156792910 |
| MycoAlert Plus Kit | Lonza, Basel, Switzerland | LT07-703 |

**Table S2. Antibodies used in the study.**

| Names | Application | Company | Category No. |
| --- | --- | --- | --- |
| Anti-IFITM1 antibody | IHC, WB | Abcam, Cambridge, MA, UK | ab224063 |
| Anti-Ki 67 antibody | IHC | Abcam, Cambridge, MA, UK | Ab16667 |
| Anti-β actin antibody | WB | Abcam, Cambridge, MA, UK | Ab8227 |
| Anti-STAT3 antibody | WB, IHC | Abcam, Cambridge, MA, UK | ab76315 |
| Anti-Cleaved Caspase 3 antibody | WB | CST, New Jersey, USA | D175 |
| Anti-Caspase 3 antibody | WB | CST, New Jersey, USA | D3R6Y |
| Anti-Caspase 7 antibody | WB | CST, New Jersey, USA | 1C12 |
| Anti-mmp 2 antibody | WB | Abcam, Cambridge, MA, UK | ab37105 |
| Anti-mmp 9 antibody | WB | Abcam, Cambridge, MA, UK | ab38898 |
| Anti-p53 antibody | WB | CST, New Jersey, USA | 7F5 |
| Anti-p21 antibody | WB | CST, New Jersey, USA | 12D1 |
| CD44 | FC | BD Biosciences, San Jose, CA, USA | 555479 |
| CD45 | FC | BD Biosciences, San Jose, CA, USA | 561866 |
| CD73 | FC | BD Biosciences, San Jose, CA, USA | 550257 |
| CD90 | FC | BD Biosciences, San Jose, CA, USA | 561970 |
| CD105 | FC | BD Biosciences, San Jose, CA, USA | 560839 |

| IHC, Immunohistochemistry; WB, Western blot; FC, Flow cytometry. |
| --- |
